# Typhoid Fever in South Africa

**Published:** 1901-03-23

**Authors:** 


### TYPHOID FEVER IN SOUTH AFRICA.

The discussion on typhoid fever in South Africa which was opened at the last meeting of the Clinical Society when Dr. H. H. Tooth detailed his personal experiences of the disease at the Modder and at Bloemfontein, leaves a strong impression on one's mind that the only means by which it will ever be possible to prevent the spread of this fever among troops so situated as ours were in Africa, must be by dealing with the infection at its very source, and making much more complete provision than has hitherto been attempted for the immediate disinfection of all excreta in standing camps.

In discussing the various agencies which were at work in causing the wide dissemination of the disease, Dr. Tooth considers, as the four chief factors, water, atmospheric disturbances, flies, and the effects of other diseases antecedent to infection. As regards water he speaks of the practical difficulties in the way of filtering or in other ways purifying the thick muddy water from the Modder, and describes the impurity of the water from other sources. Water, however, was far from being the only, or the chief, mode of dissemination of the poison. The great sandstorms and the more localised whirlwinds, which earned for themselves the name of "sand-devils," kept everything covered with dust, and in proportion as the sand became contaminated with fæcal and urinary matters, these matters also were deposited broadcast over and into the food and drink. As a factor in the spread of enteric fever we must, then, by no means overlook the sandstorms which are everyday phenomena in many parts of the world where the British Army may be sent, and it is quite a question whether they have not been a more important factor in this war than water itself. That the water contained typhoid bacilli is a matter of speculation, but that enteric stools and urine must have been deposited in the latrines and other places by men in the early stages of the disease there can be no reasonable doubt, any more than there can be of the dust derived therefrom being widely scattered by atmospheric influences.

As may be expected the conditions in the large camps were particularly favourable to the growth and multiplication of flies, which soon became a terrible pest. They were all over the food, and the roofs of the tents were black with them. It is not unreasonable, then, to regard them as a very possible agency in spreading the disease, and that they were so is borne out by the fact that with the first appearance of frost enteric fever rapidly disappear. Considering the warmth of the days even in the winter it is hardly credible that the almost sudden cessation of an epidemic could be due to the effect of cold upon the enteric-fever bacilli. But flies are extremely sensitive to change of temperature, and the cold nights kill them off rapidly, and on consideration of these points it is surely justifiable to assign an important share to flies in the spreading of infection.

Dr. Tooth makes the important suggestion that enteric-fever bacilli in the blood and tissues of persons more or less immune to them, may be roused into activity by the presence of other pathogenic organisms. This is fully in accordance with Sanarelli's experiments, and Dr. Tooth suggests that the occurrence of an attack of simple gastro-enteritis might thus determine the occurrence of an attack of typhoid fever in a patient in whom the typhoid bacilli might otherwise have remained inert. He says that the enteric-fever epidemic so overshadowed in the public mind all other medical ailments that we were apt to overlook the fact that dysentery, or its milder forms which were included under the head of diarrhœa, was quite as prevalent among the troops as enteric fever was, and he urges that in view of what we know about the manner in which enteric-fever bacilli may be roused to virulence by the co-operation of other organisms, diarrhœa assumes a new importance. A widely distributed and almost universal dysenteric diarrhœa, together with a daily exposure to infection by enteric bacilli, provide the elements necessary for a severe epidemic of enteric fever. As a fact many cases of enteric fever did occur in the course of attacks of dysentery.

Such are some of the points set forth with much cogency by Dr. Tooth as indicating the factors involved in the dissemination of the disease, and if we consider for a moment what these things mean we become very hopeless of warding off the disease by measures aimed solely at preventing the access of an almost universally disseminated poison. To submit to pains and discomforts for the sake of pure water, while all the time we are eating dirt, is obviously misspent zeal; to waste our energies in trying to keep out the dust while flies swarm around and deposit fresh fæcal matter on our very food is labour lost. The only chance is to begin at the beginning and ensure that what the dust brings, what the flies carry, and what the water contains, however dirty it may look, shall at least not contain typhoid infection; and this we can only do by taking care that the very source and origin of the poison, namely, the excreta, shall be disinfected from the first—not the excreta of the sick alone, but of all, for we cannot tell until the onset of the disease who is healthy and who may be the bearer of infection. Such an undertaking is no doubt difficult, and on the march is obviously impossible. But it is in standing camps that the mischief arises, and in standing camps the appliances necessary for the purpose and the labour involved in their proper employment would probably be far less than the appliances required and the labour involved in the treatment of an outbreak of enteric fever—the natural, and apparently the inevitable, penalty of neglect in this matter.

